# The Blind Spot of Bone Disease: Adult Hypophosphatasia in a Middle-Aged Man With Prolonged Fracture Healing

**DOI:** 10.7759/cureus.119345

**Published:** 2026-11-09

**Authors:** Albatool A Busbaih, Alkawthar A Busbaih, Salma Hussein, Abdullah Alfaraj

**Affiliations:** 1 Endocrinology, King Fahad Specialist Hospital, Dammam, SAU; 2 Medicine and Surgery, Imam Abdulrahman Bin Faisal University, Dammam, SAU

**Keywords:** alkaline phosphatase, alpl gene, asfotase alfa, bone mineralization, delayed fracture healing, hypophosphatasia

## Abstract

Hypophosphatasia (HPP) is a rare inherited disorder caused by pathogenic variants in the ALPL gene, which encodes tissue-nonspecific alkaline phosphatase. In adults, it presents with nonspecific musculoskeletal features and is often mistaken for osteoporosis. We describe a 47-year-old man referred to endocrinology with a previous diagnosis of osteoporosis and recurrent femoral fractures with markedly delayed healing, at ages 30 and 39 years. He also had chronic musculoskeletal pain and atraumatic loss of teeth early in childhood, at age four; a bone scan showed multiple metatarsal fractures, and nephrocalcinosis was present. Serum alkaline phosphatase was persistently low and pyridoxal 5′-phosphate was elevated at 494 mcg/L (reference range: 5-50 mcg/L), while thyroid, parathyroid, cortisol, pituitary, renal, hepatic, and vitamin D evaluations were unremarkable. Several relatives had related manifestations. Adult HPP was diagnosed clinically in 2017, and genetic testing later identified an apparently homozygous, likely pathogenic ALPL variant (c.214A>G, p.Ile72Val). Treatment with asfotase alfa was associated with marked improvement, and he regained the ability to walk without a cane. This case shows that persistently low alkaline phosphatase in an adult with recurrent fractures and poor fracture healing should prompt evaluation for HPP before osteoporosis is assumed and that the diagnosis need not wait for genetic confirmation. Greater clinical awareness may shorten diagnostic delay.

## Introduction

Hypophosphatasia (HPP) is a rare inherited disorder of bone and mineral metabolism caused by loss-of-function variants in the ALPL gene, which result in deficient tissue-nonspecific alkaline phosphatase (TNSALP) activity and impaired mineralization of bone and teeth. Deficient TNSALP activity leads to the accumulation of its natural substrates, including inorganic pyrophosphate, a potent inhibitor of bone mineralization, and pyridoxal 5′-phosphate (PLP), the main circulating form of vitamin B6. The biochemical hallmark of HPP is therefore persistently low serum alkaline phosphatase (ALP) activity with elevated PLP, a pattern that distinguishes it from osteoporosis, in which ALP is typically normal or elevated [1].

HPP may be inherited in an autosomal recessive or autosomal dominant pattern. Autosomal recessive inheritance, in which both copies of the ALPL gene carry pathogenic variants, is generally associated with the more severe perinatal and infantile forms, whereas milder forms, including many adult cases, may result from either two variants or a single variant with a dominant-negative effect. As a result, disease expression varies widely, even within the same family. The clinical spectrum ranges from perinatal lethal forms to odontohypophosphatasia, which affects only the teeth, and adult HPP. Non-genetic factors may also affect outcomes; in particular, bisphosphonates given for presumed osteoporosis may be harmful in unrecognized HPP [1].

In adults, the presentation is nonspecific, and the disease is frequently mistaken for osteoporosis. We report a man with adult HPP whose recurrent femoral fractures and delayed healing were attributed to osteoporosis for years, to highlight the diagnostic clues and the consequences of delayed recognition.

## Case presentation

A 47-year-old man was referred to the endocrinology service following a previous diagnosis of osteoporosis and a history of recurrent femoral fractures with markedly delayed healing. His first fracture occurred at the age of 30 following a fall from standing height and required more than seven months to heal. At the age of 39, he sustained a second femoral fracture, a left subtrochanteric fracture, also following a fall from standing height, with healing taking nearly one year. Both fractures resulted from low-energy trauma and were therefore considered fragility fractures. Radiographs show intramedullary nails in both femurs (Figure 1A), and a lateral view of the right lower leg shows an unhealed tibial fracture of unknown cause (Figure 1B, arrow). Despite previous orthopedic management, the underlying cause of his recurrent fractures and delayed healing remained unexplained, prompting further endocrinological evaluation.

**Figure 1:**
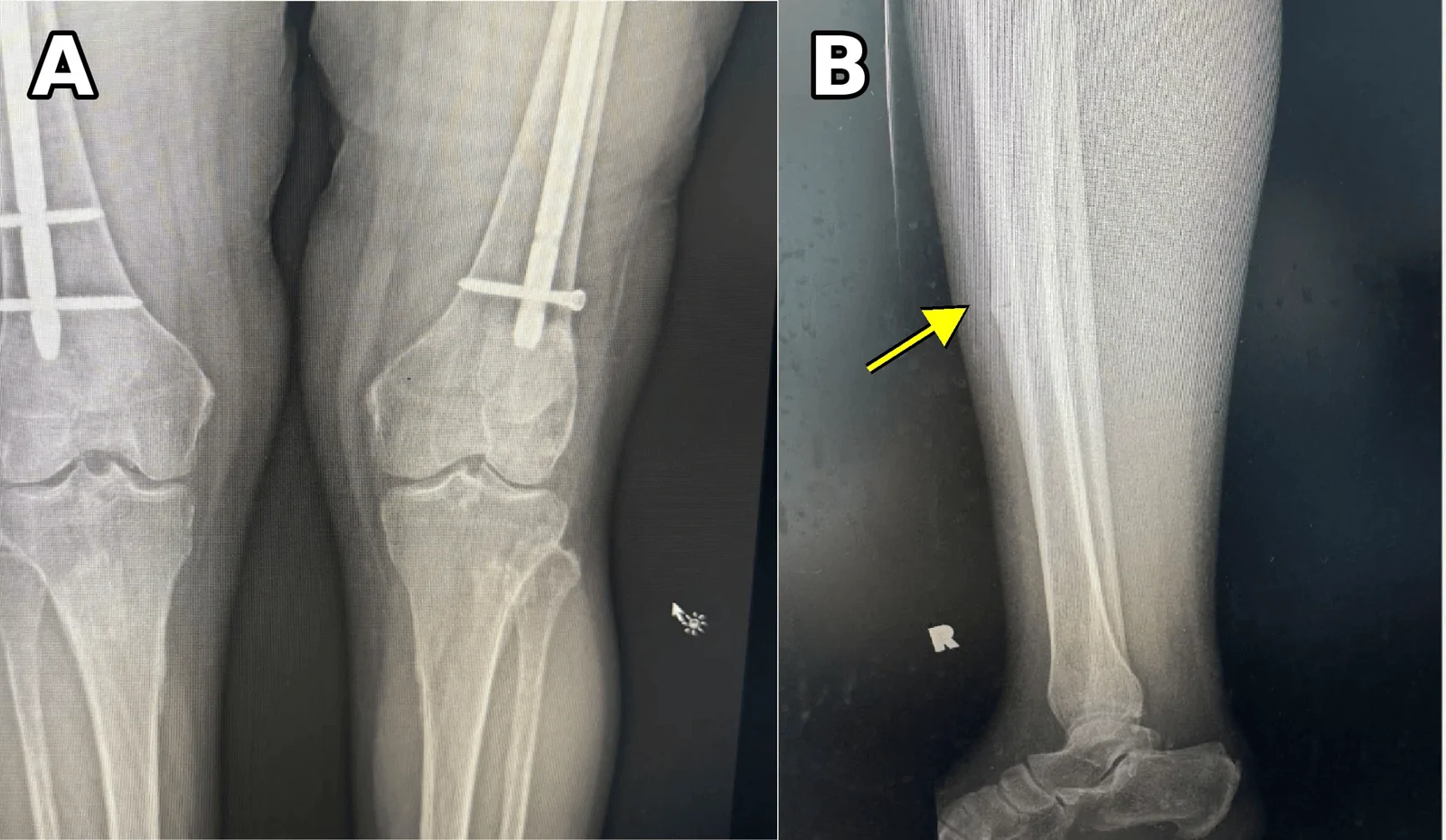
Radiographs of the patient obtained in May 2025. (A) Anteroposterior radiograph of both knees and distal femurs showing intramedullary nails with distal locking screws in both femurs. (B) Lateral radiograph of the right lower leg and ankle showing an unhealed fracture of the tibia of unknown cause (arrow).

A detailed medical history revealed chronic musculoskeletal pain, atraumatic loss of teeth early in childhood (at age four), and prolonged fracture healing. A bone scan confirmed multiple metatarsal fractures, providing additional evidence of skeletal involvement. Nephrocalcinosis was identified on computed tomography. His symptoms substantially affected his mobility, independence, social life, and overall quality of life, eventually necessitating the use of a walking cane.

Biochemical evaluation demonstrated persistently low serum ALP activity and an elevated serum pyridoxal 5′-phosphate concentration of 494 mcg/L (reference range: 5-50 mcg/L). Serum ALP was persistently low on repeated testing (10 and 18 U/L; reference range: 40-129 U/L). Further investigations showed thyroid-stimulating hormone, cortisol, parathyroid hormone, and vitamin D levels within their respective reference ranges. Renal function was stable, with creatinine within the patient’s baseline and no electrolyte abnormalities. Liver enzymes, complete blood count, and the assessed pituitary hormonal profile, including testosterone, were unremarkable. Serum protein electrophoresis was negative, and the remaining bone profile was unremarkable. These findings, together with the persistently low ALP, elevated pyridoxal 5′-phosphate, recurrent and poorly healing fractures, multiple metatarsal fractures, chronic musculoskeletal pain, early atraumatic tooth loss, and nephrocalcinosis, supported the diagnosis of HPP after consideration of alternative explanations for low ALP.

A detailed family history revealed related clinical and skeletal manifestations among several relatives, although the presentation varied between family members. Three of his four brothers and one of his two sisters had a similar presentation, and his affected sister is receiving asfotase alfa. Genetic testing performed in the patient and some family members provided additional evidence supporting familial HPP. The patient was diagnosed clinically with adult HPP in 2017, before molecular confirmation. Genetic testing was performed by sequence analysis of the ALPL gene on a peripheral blood sample at an accredited external laboratory (CGC Genetics) and identified a c.214A>G (p.Ile72Val) variant, detected in apparent homozygosity and classified as likely pathogenic.

Following diagnosis, the patient received asfotase alfa, a recombinant enzyme replacement therapy, at a dose of 6 mg/kg/week, given as 2 mg/kg three times weekly; the exact start date was not available. Treatment was associated with marked clinical improvement. His mobility improved sufficiently for him to walk without a cane, representing a meaningful improvement in functional independence and quality of life. The prolonged diagnostic delay had previously contributed to psychological distress and adversely affected his social and occupational functioning.

## Discussion

Adult HPP is a rare inherited disorder of bone and mineral metabolism caused by pathogenic variants in the ALPL gene, which encodes tissue-nonspecific alkaline phosphatase. Reduced enzyme activity leads to the accumulation of inorganic pyrophosphate, an inhibitor of hydroxyapatite formation, resulting in impaired bone and dental mineralization [1]. The clinical manifestations of adult HPP are heterogeneous and may overlap with those of osteoporosis, osteomalacia, and chronic musculoskeletal pain syndromes, contributing to diagnostic challenges and prolonged delays in recognition [2-5].

Several conditions should be considered in the differential diagnosis of adult HPP. Osteoporosis is the most common misdiagnosis; however, serum ALP is typically normal or elevated in osteoporosis, and metatarsal stress fractures, early tooth loss, and markedly delayed fracture healing are not typical features. Osteomalacia due to vitamin D deficiency or hypophosphatemic disorders may also present with bone pain, pseudofractures, and poor fracture healing, but is usually associated with elevated rather than low ALP, together with abnormalities in vitamin D, calcium, phosphate, or parathyroid hormone. Chronic pain syndromes and calcium pyrophosphate deposition disease may also overlap with adult HPP, particularly in patients who present mainly with musculoskeletal pain [1,3].

The present case illustrates several characteristic manifestations of adult HPP, including recurrent femoral fractures, markedly delayed fracture healing, multiple metatarsal fractures, chronic musculoskeletal pain, and atraumatic loss of teeth early in childhood (at age four years). Despite the presence of these features, the patient was initially labeled as having osteoporosis. The recurrence of fractures and the prolonged healing of both femoral fractures should have prompted earlier investigation for an underlying mineralization disorder. This case emphasizes the importance of reconsidering an initial diagnosis when the clinical course is inconsistent with uncomplicated osteoporosis. These features are consistent with published adult HPP series, in which fractures, particularly metatarsal stress fractures, musculoskeletal pain, and premature tooth loss are among the most frequently reported manifestations [2,5].

Persistently low serum ALP is a key diagnostic clue in HPP. However, low ALP is not specific to this condition and requires evaluation for alternative causes, including nutritional deficiencies (such as malnutrition and zinc or magnesium deficiency), relevant medications (such as antiresorptive agents and glucocorticoids), endocrine disorders (such as hypothyroidism), and other systemic conditions (such as celiac disease, multiple myeloma, and Wilson disease) [3,5]. In the present case, the evaluation showed unremarkable thyroid function, cortisol, parathyroid hormone, vitamin D, renal function, liver enzymes, complete blood count, and the assessed pituitary hormonal profile, including testosterone. Serum protein electrophoresis was negative, and no electrolyte abnormalities were identified. The elevated pyridoxal 5′-phosphate concentration of 494 mcg/L, together with the characteristic skeletal and dental manifestations, strongly supported HPP. These findings illustrate the importance of interpreting low ALP in the context of the complete clinical and biochemical picture rather than attributing recurrent fractures and delayed healing to osteoporosis alone.

The diagnosis was established clinically in 2017, before subsequent genetic testing identified an apparently homozygous ALPL c.214A>G (p.Ile72Val) variant, classified as likely pathogenic. The adult diagnostic criteria proposed by the HPP International Working Group apply to patients with persistently low ALP without another identifiable cause, and require either two major criteria or one major and two minor criteria [6]. This patient met three major criteria (a likely pathogenic ALPL variant, elevated pyridoxal 5′-phosphate as a natural substrate, and recurrent metatarsal fractures) and four minor criteria (poorly healing fractures, chronic musculoskeletal pain, early atraumatic tooth loss, and nephrocalcinosis), as shown in Table 1. The clinical and biochemical findings were supported by the genetic results, illustrating that molecular confirmation can strengthen diagnostic certainty but should not delay recognition when the clinical presentation is compelling. Genetic assessment is also important for family counseling and for clarifying the inheritance pattern, particularly when several relatives have related manifestations.

**Table 1:** Adult hypophosphatasia diagnostic criteria and the patient's findings. Criteria of the HPP International Working Group for adults; the criteria apply to persistently low ALP without another identifiable cause, and diagnosis requires two major criteria, or one major and two minor criteria [6]. ALP, alkaline phosphatase; ALPL, alkaline phosphatase, biomineralization associated gene.

Criterion	Status in this patient	Patient's findings
Prerequisite: persistently low ALP without another identifiable cause	Present	Persistently low serum ALP; other causes evaluated and not found
Major: pathogenic or likely pathogenic ALPL variant	Present	ALPL c.214A>G (p.Ile72Val), apparent homozygosity, classified as likely pathogenic
Major: elevation of natural substrates	Present	Pyridoxal 5′-phosphate 494 mcg/L (reference range: 5-50 mcg/L)
Major: recurrent metatarsal fractures	Present	Multiple metatarsal fractures on bone scan
Major: atypical femoral fractures (pseudofractures)	Not established	Two femoral fragility fractures after falls from standing height, including a left subtrochanteric fracture; atypical features not documented
Minor: poorly healing fractures	Present	Femoral fractures healed after more than seven months and nearly one year
Minor: chronic musculoskeletal pain	Present	Chronic musculoskeletal pain
Minor: early atraumatic loss of teeth	Present	Atraumatic loss of teeth early in childhood, at four years
Minor: chondrocalcinosis	Not documented	Not reported in the available records
Minor: nephrocalcinosis	Present	Nephrocalcinosis

Notably, the same ALPL c.214A>G (p.Ile72Val) variant has previously been reported in the homozygous state in an infant with HPP who presented with pseudotumor cerebri [7]. In contrast, our patient carries the same variant in apparent homozygosity but presented with the adult form of the disease. This comparison illustrates the marked phenotypic variability of HPP, in which the same genotype may be associated with different ages of onset and clinical severity.

The family history provided an additional diagnostic clue. Several family members had similar or related clinical problems, although the manifestations differed between individuals. Genetic testing in the patient and some relatives supported familial HPP. Variable clinical expression can complicate recognition of inherited disorders, and a detailed family history may identify affected relatives who have not previously been evaluated. Appropriate clinical assessment and genetic counseling should therefore be considered for family members when indicated.

The diagnostic challenges encountered in this case are consistent with international reports describing substantial delays in HPP diagnosis. Högler et al. reported that diagnostic delay is common among patients with HPP, while Brandi et al. highlighted the challenges of establishing the diagnosis in adults, in whom symptoms may be nonspecific and overlap with more common musculoskeletal conditions [3,4]. Delayed recognition may prolong symptoms, postpone appropriate treatment, and expose patients to potentially inappropriate management for presumed osteoporosis.

The epidemiology of HPP in Saudi Arabia also deserves attention. Although the disease has been reported locally, its true prevalence in the Saudi population remains unknown. A recent multicenter Saudi cohort described 19 patients with clinically and genetically confirmed HPP from six centers, with perinatal, infantile, or childhood onset and no separately reported adult-onset disease, demonstrating that the disease is present in the Kingdom and that its clinical and genetic manifestations are heterogeneous [8]. However, this cohort was not designed to establish population-based prevalence. Consequently, the limited number of published cases should not be interpreted as evidence that HPP is exceptionally uncommon in Saudi Arabia; underrecognition and the absence of comprehensive epidemiological data may contribute to the apparent scarcity of reported cases. Further population-based research is needed to clarify disease frequency and improve understanding of its clinical spectrum in the region.

Limited awareness of HPP among healthcare professionals may further contribute to delayed diagnosis, particularly in adults initially evaluated for osteoporosis, recurrent fractures, chronic musculoskeletal pain, or dental problems. This case highlights the need for greater education and awareness among endocrinologists, orthopedic surgeons, primary care physicians, and other clinicians involved in the assessment of metabolic bone disease. Educational initiatives should emphasize the significance of persistently low ALP and encourage clinicians to investigate characteristic skeletal and dental manifestations before assigning a diagnosis of osteoporosis. Earlier recognition may facilitate timely specialist referral, appropriate management, family assessment, and genetic counseling, while reducing the physical, psychological, social, and occupational burden associated with prolonged diagnostic uncertainty. A practical screening approach is to review serum ALP in all adults evaluated for osteoporosis or recurrent fractures and to measure PLP in those with persistently low ALP before starting antiresorptive therapy [3,6].

An additional consideration is the potential harm associated with inappropriate osteoporosis-directed treatment in patients with unrecognized HPP. Antiresorptive agents, particularly bisphosphonates, warrant special caution because of concerns regarding their use in this condition and the association between HPP and atypical femoral fractures [1,3]. In the present case, the previous diagnosis of osteoporosis underscores the importance of investigating persistently low ALP before initiating or escalating osteoporosis therapy. However, the available records do not establish the precise osteoporosis medication administered or demonstrate that a treatment-related complication occurred. Therefore, the possibility of inappropriate treatment should be discussed as a clinical concern rather than as a proven adverse outcome in this patient.

The patient’s clinical response to asfotase alfa is another important feature of this report. Following enzyme replacement therapy, he demonstrated marked functional improvement and regained the ability to walk without a cane. This improvement was associated with better mobility and quality of life after years of physical and psychosocial burden. Although the response in a single patient cannot establish treatment efficacy in a broader population, it illustrates the potential importance of recognizing HPP and considering disease-specific treatment in appropriately selected patients.

This report has limitations. It describes a single patient, and some historical laboratory, treatment, and longitudinal outcome details were not available for review. Further documentation of segregation of the variant among affected relatives and objective measures of functional improvement would strengthen the report. Nevertheless, this case emphasizes the importance of integrating clinical, biochemical, radiological, and familial information to recognize adult HPP, reduce diagnostic delay, and support appropriate management.

## Conclusions

Adult HPP is an important but frequently overlooked cause of recurrent fractures, delayed fracture healing, metatarsal fractures, early atraumatic tooth loss, and chronic musculoskeletal pain. Persistently low serum ALP should prompt further evaluation, including assessment of relevant biochemical markers, consideration of alternative causes, and genetic testing when appropriate. A detailed family history may provide additional clues and support the identification of other affected relatives.

This case illustrates the consequences of delayed diagnosis, including impaired mobility and substantial psychological, social, and occupational burden. The patient’s marked improvement following asfotase alfa treatment, including regaining the ability to walk without a cane, highlights the potential value of timely recognition and appropriate disease-specific management. In Saudi Arabia, greater clinical awareness, educational initiatives, and further epidemiological research are needed to improve recognition of HPP and reduce delays in diagnosis and treatment.
